# Two New Computational Methods for Universal DNA Barcoding: A Benchmark Using Barcode Sequences of Bacteria, Archaea, Animals, Fungi, and Land Plants

**DOI:** 10.1371/journal.pone.0076910

**Published:** 2013-10-18

**Authors:** Akifumi S. Tanabe, Hirokazu Toju

**Affiliations:** 1 Graduate School of Global Environmental Studies, Kyoto University, Kyoto, Kyoto, Japan; 2 Research Center for Aquatic Genomics, National Research Institute of Fisheries Science, Fisheries Research Agency, Yokohama, Kanagawa, Japan; 3 Graduate School of Human and Environmental Studies, Kyoto University, Kyoto, Kyoto, Japan; Consiglio Nazionale delle Ricerche (CNR), Italy

## Abstract

Taxonomic identification of biological specimens based on DNA sequence information (a.k.a. DNA barcoding) is becoming increasingly common in biodiversity science. Although several methods have been proposed, many of them are not universally applicable due to the need for prerequisite phylogenetic/machine-learning analyses, the need for huge computational resources, or the lack of a firm theoretical background. Here, we propose two new computational methods of DNA barcoding and show a benchmark for bacterial/archeal 16S, animal *COX1*, fungal internal transcribed spacer, and three plant chloroplast (*rbcL*, *matK*, and *trnH*-*psbA*) barcode loci that can be used to compare the performance of existing and new methods. The benchmark was performed under two alternative situations: query sequences were available in the corresponding reference sequence databases in one, but were not available in the other. In the former situation, the commonly used “1-nearest-neighbor” (1-NN) method, which assigns the taxonomic information of the most similar sequences in a reference database (i.e., BLAST-top-hit reference sequence) to a query, displays the highest rate and highest precision of successful taxonomic identification. However, in the latter situation, the 1-NN method produced extremely high rates of misidentification for all the barcode loci examined. In contrast, one of our new methods, the query-centric auto-*k*-nearest-neighbor (QCauto) method, consistently produced low rates of misidentification for all the loci examined in both situations. These results indicate that the 1-NN method is most suitable if the reference sequences of all potentially observable species are available in databases; otherwise, the QCauto method returns the most reliable identification results. The benchmark results also indicated that the taxon coverage of reference sequences is far from complete for genus or species level identification in all the barcode loci examined. Therefore, we need to accelerate the registration of reference barcode sequences to apply high-throughput DNA barcoding to genus or species level identification in biodiversity research.

## Introduction

Biodiversity surveys are important when formulating policies for the conservation of endangered species, assessing the environmental impacts of land development projects, and exploring novel bioproducts [1], [2]. In biodiversity surveys, taxonomic identification of collected organismal specimens is a major bottleneck process. Taxonomic identification based on DNA sequences (a.k.a. DNA barcoding or molecular identification) is promising in that it enables the application of standardized and high-throughput taxonomic identification protocols in biodiversity research [3]–[8]. Given that traditional taxonomic identification based on morphology is often difficult for species-rich lineages of soil fungi, marine/freshwater plankton, and prokaryotes, DNA barcoding offers an alternative or supplemental research approach for the description and identification of these microorganisms [9]–[12]. Moreover, because extracellular DNA released into soil or water can be PCR-amplified and/or sequenced [13], the DNA barcoding of such “environmental DNA” dissolved in water potentially enables ultrarapid surveys of aquatic macroorganisms in a lake [14], [15]. Consequently, such recent technical developments and the declining cost of DNA sequencing have increased the opportunities to utilize DNA barcoding in ecological and evolutionary studies [13], [16], [17]. However, the development of a theoretically firm framework to “translate” raw DNA sequencing data into organismal taxonomic information is crucial (see Coissac et al. [18] and references therein).

The existing methods for inferring organismal taxonomy based on DNA sequencing data are classified into four categories, i.e., “tree-based,” “composition-based,” and “similarity-based” approaches, and their hybrids. These approaches vary in their requirements for reference database information, prerequisite phylogenetic or machine-learning analyses, and their potential taxonomic range of application. In the tree-based approach, the taxonomy of an operational taxonomic unit (OTU) of a query is inferred by placing the OTU within a given reference phylogenetic tree as implemented in software such as MLTreeMap [19] and pplacer [20]. In the composition-based approach, a query sequence is assigned to a taxonomic unit based on the pattern-recognition of a *k*-mer-length word composition as implemented in PhyloPythiaS [21], TACOA [22], and RDPClassifier [23]. In this approach, the word composition of reference sequences needs to be learned by the programs before performing the taxonomic assignment of a query sequence. In contrast to these two approaches that need prerequisite phylogenetic or word-composition analyses, the similarity-based approach requires only raw reference sequences with taxonomic information that is available in public nucleotide databases. Conducting nucleotide (or protein) BLAST searches [24] and taxonomic assignment manually (i.e., with users' eyes) or by using MEGAN [25] is the most commonly used method in this approach. SOrt-ITEMS [26] and CARMA3 [27] are also based on BLAST searches and enable automated BLAST search and taxonomic assignment using their own similarity cutoff. BRONX [28] also uses a similarity-based method based on a unique search engine for similar sequences. In particular, MEGAN assigns a query sequence to the lowest taxonomic level common to the BLAST-hit database sequences that are similar to the query sequence (lowest common ancestor [LCA] algorithm [25]). The hybrid approaches of taxonomic assignments include a combination of similarity-based and tree-based approaches as implemented in SAP [29] or a combination of similarity-based and composition-based approaches as provided in PhymmBL [30]. For example, “Barcoder” and “ConstrainedNJ” algorithms implemented in SAP [29] first conduct similar sequence retrievals from a reference database using BLAST, and the multiple sequence alignment of a query and the retrieved sequences is subsequently performed. Those programs then place the query OTU within Bayesian [31] or neighbor-joining [32] phylogenetic trees.

As the size of public nucleotide databases is growing rapidly, one of the most important measures for choosing among the existing taxonomic assignment methods is the ability to handle huge reference sequence databases. The similarity-based method is therefore promising because it is less computationally intensive in such preprocessing stages as prerequisite phylogenetic/word-composition analyses and database construction.

To further explore the possibility of the use of a similarity-based approach in high-throughput DNA barcoding, the theoretical background of the approach needs to be rigorously investigated. In commonly used similarity-based barcoding programs such as MEGAN [25], users are required to set arbitrary BLAST-search parameters. For example, in the *n*%-nearest-neighbor (*n*%-NN) approach, the parameter *n* designates the minimum cutoff identity in the retrieval of reference database sequences that are similar to a query. The *n*%-identical reference sequences are then processed by the LCA algorithm, wherein the taxonomic unit common to all the *n*%-identical sequences is assigned to the query at a taxonomic level as low as possible (e.g., genus; [25]). An alternative parameter, *k*, is similarly used in the retrieval of the *k*-most similar reference sequences to a query sequence (*k*-nearest-neighbor [*k*-NN] approach). However, a simple question arises at this point: how large *n*/*k* value should be given? In an extreme situation, wherein the reference sequences of all potentially observable species are available in a reference database, the setting 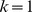 is expected to return the best result. However, reference nucleotide databases are far from complete in most organismal taxa (e.g., [11]), and hence the optimal *n* or *k* values should differ among queries. Thus, we need to develop a generalized theoretical criterion that enables us to choose optimal *k* or *n* values for each query.

In this study, we propose two new DNA barcoding methods that are based on a firm criterion for searching similar sequences. After describing the details of the new methods, we conducted an intensive benchmarking exercise using publicly available database sequences of bacterial/archeal 16S, animal *COX1*, fungal internal transcribed spacer (ITS), and three plant chloroplast (*rbcL*, *matK*, and *trnH*-*psbA*) barcode loci. The two methods, nearest-neighbor-centric auto-*k*-NN (NNCauto) and query-centric auto-*k*-NN (QCauto), provided an intuitive theoretical background for the DNA barcoding of all types of organisms and genetic loci, i.e., they are universally applicable. As the methods were developed with the aid of the similarity-based approach, the fast processing of large data sets was possible. Moreover, the new methods are characterized by consistently low misidentification rates in the DNA barcoding of various organismal groups. We present the results of a benchmark of several existing methods and our new methods, and thereby review the characteristics of those methods.

## Materials and Methods

### Developing and Implementing the New Methods

A previous study has proposed a criterion for choosing an optimal *n*/*k* value [33]. Under the proposed “all species barcodes” criterion, “the maximum genetic distance between a query sequence and the reference barcode sequences of the resulting (output) taxonomic unit needs to be smaller than the minimum distance between the query sequence and the reference barcode sequences of all the other taxonomic units” [33]. This criterion is intuitive, but is computationally intensive in the calculation of the maximum distance between a query and the reference barcode sequences of the resulting taxonomic unit. This criterion often fails taxonomic assignment, especially when the presence of the DNA sequences that lack barcode regions (i.e., non-barcode sequences) in a database inflates the maximum genetic distance between a query and the reference sequences of the resulting taxonomic unit.

**Figure 1:**
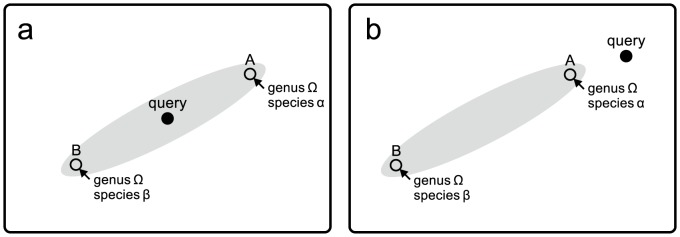
Schematic illustration of the relationship between query and reference sequences. A query sequence (filled circle) and reference sequences similar to the query sequence (open circle) are shown. The range of nucleotide variation of the genus 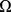 (gray area) is shown with reference sequences of species 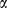 and 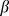 in the genus (A and B, respectively). Distance between the sequences represents genetic distance in the schematic two-dimensional space. (a) A case in which our new criterion works well. The query falls within the nucleotide variation range of genus 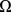. (b) A case in which our new criterion might produce misidentification. Because the genetic distance between a query sequence and the sequence similar to it (A) is smaller than the genetic distance between sequence A and sequence B, the query sequence will be assigned to the genus 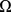 under our new criterion.

To solve these problems, we herein propose a new criterion. The criterion met when “the maximum genetic distance among the reference barcode sequences of the resulting taxonomic unit is larger than the minimum genetic distance between a query sequence and the reference barcode sequences of all taxonomic units” (Fig. 1). As shown below, we can examine whether this criterion is fulfilled without undertaking a computationally intensive calculation of the maximum genetic distance among the reference barcode sequences of resulting taxonomic units. Thus, the new methods proposed here are much less computationally intensive than “all species barcodes” and are free of the influence of the contamination of non-barcode sequences in reference sequence databases.

#### Nearest-Neighbor-Centric Auto-*k*-Nearest-Neighbor (NNCauto) Method

**Figure 2:**
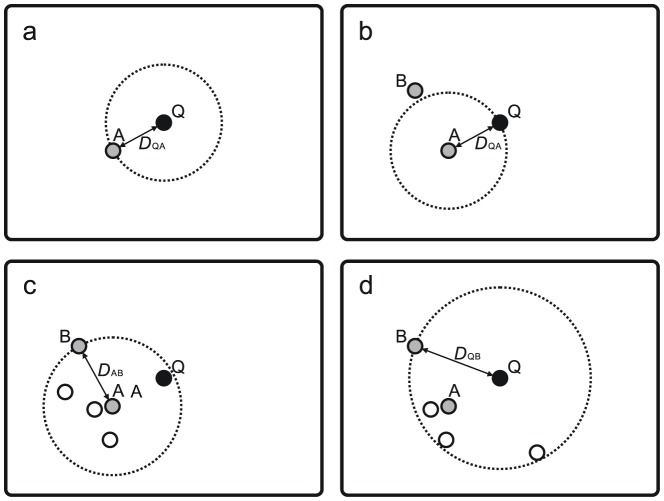
Schematic illustration of the NNCauto and QCauto methods. The processes of the NNCauto method are summarized as follows: (a) By a BLAST-search of a query sequence (Q), a nearest-neighbor sequence (A) is retrieved. (b) By a BLAST-search of A, a borderline sequence (B) is retrieved. (c) By an additional BLAST-search of A, all neighborhood sequences (open circles) are retrieved. Finally, the query is identified at the lowest taxonomic level where the taxonomic information of all the neighborhood sequences including A and B is consistent with each other (i.e., lowest common ancestor algorithm [21]). In the QCauto method, the processes a and b are shared with the NNCauto method, but neighborhood sequences are retrieved by a BLAST-search of Q (d). After the search of neighborhood sequences, the query is identified by the LCA algorithm as in the NNCauto method. A bidirectional arrow indicates genetic distance between two sequences, and a dotted circle represents the range of nucleotide variation that meets the requirement of a BLAST-search.

The implementation of the NNCauto method can be summarized in four steps. First, reference database sequences similar to a query sequence (Q) were BLAST-searched, and then the nearest-neighbor sequence (A) and its distance (BLAST raw score) to a query sequence (hereafter, 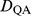) was obtained (step I; Fig. 2a). Second, reference sequences similar to A were also BLAST-searched, and the “borderline sequence” (B), whose distance to A (hereafter, 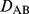) was smallest in the sequences that were farther from A than Q (i.e., 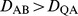), was obtained (step II; Fig. 2b). Third, a BLAST-search of reference sequences similar to A was performed again, and then all the “neighborhood sequences” (hereafter, Ns) whose distance to A was equal to or smaller than 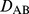 were retrieved (i.e., 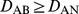, where 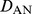 represents the distance between A and an N; step III; Fig. 2c). Finally, a taxonomic unit was assigned to the query at the lowest taxonomic level where the taxonomic information for all of the nearest-neighbor (A), borderline (B), and neighborhood (N) sequences was consistent (i.e., LCA algorithm; step IV). When multiple nearest-neighbor sequences existed, the borderline (B) and neighborhood (N) sequences were searched for each nearest-neighbor sequence (A), and all of the nearest-neighbor (A), borderline (B), and neighborhood (N) sequences were used in the LCA process. Because 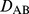 is equal to or smaller than the maximum genetic distance among the reference barcode sequences of the resulting (output) taxonomic unit, the new criterion mentioned above was fulfilled by this method and the query sequence was expected to fall within the nucleotide variation range of the resulting taxonomic unit (Fig. 1).

#### Query-Centric Auto-*k*-Nearest-Neighbor (QCauto) Method

In the QCauto method, steps I, II, and IV are the same as in the NNCauto method. In step III of the QCauto method, the distance between a query (Q) and borderline (B) sequences (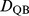) was calculated. A BLAST-search of reference sequences similar to the query was then performed, and subsequently, all the neighborhood sequences (Ns) whose distance to the query sequences was equal to or smaller than 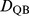 were retrieved (i.e., 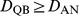; Fig. 2d). If multiple borderline (B) sequences existed, the borderline sequence that was closest to the query (Q) sequence was used in step III. Because 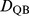 is equal to or smaller than the maximum genetic distance among the reference barcode sequences of the resulting taxonomic unit, the new criterion was also fulfilled by this method, and the query sequence was expected to fall within the range of nucleotide variation of the resulting taxonomic unit.

The process of the QCauto method is more intuitive than that of the NNCauto method in that it searches for neighborhood sequences (Ns) around a query (Fig. 2d). However, the QCauto method is expected to be slower than the NNCauto method: while the BLAST-searches in steps II and III could be integrated in the NNCauto method, the QCauto method required independent BLAST-searches in steps II and III.

#### Availability

The two new methods (NNCauto and QCauto) described above were implemented in the software package “Claident”, which is available at http://www.claident.org/ under GNU General Public License ver. 2. In addition to the NNCauto and QCauto methods, the program supports the *n*%-NN, *k*-NN, and *n*%-*k*-NN methods. The *n*%-*k*-NN method uses the *k*-most similar sequences of *n*%-identical sequences. This program requires BLAST+ [34] for the BLAST-search of nearest-neighbor (A), borderline (B), and neighborhood (N) sequences.

### Performance Benchmark of the New and the Existing Methods

#### Construction of the Reference Sequence Databases and the Taxonomy Databases

The reference sequence databases of animal *COX1* (*COI*), bacterial/archaeal 16S, fungal ITS, and *matK*, *rbcL*, and *trnH*-*psbA* spacer of land plants (Embryophyta) were constructed by the following procedure. The NCBI (http://www.ncbi.nlm.nih.gov/) GenBank nucleotide sequence database was searched using the keywords described in Table S1, and the GenBank IDs (GIs) of the matched sequences were retrieved. The GIs of the sequences that had both genus- and species-level taxonomic information were then selected in the NCBI taxonomy database (downloaded from the NCBI ftp server on May 15, 2012). These selected GIs were used to construct the local reference sequence databases of the respective barcode loci listed above. Reference sequences were extracted from the NCBI nt sequence database (downloaded from NCBI ftp server on May 11, 2012) using the selected GIs. For each local database of reference sequences, the corresponding local NCBI taxonomy database was constructed. Although we also tried to construct the reference databases of protist/algal 18S and 28S rDNA, the number of sequences obtained was too small to perform a benchmark despite their high phylogenetic diversity.

#### Selection of Query Sequences

To perform a benchmark of the new and existing DNA barcoding methods, up to 100 genera per order for land plants, and 500 genera per phylum/division for animals, bacteria/archaea, and fungi were randomly selected from the above-mentioned taxonomy databases. One sequence was then randomly selected from each of the selected genera in each organismal group.

For the benchmark of each query, results were obtained under two types of setting: the full-length sequences of queries were used under the “full-length” setting, while 200 contiguous nucleotide sites were randomly retrieved from query sequences that were used under the “mini-barcode” [35]–[37] query setting.

#### Running Benchmark

The assignment performances of the nai
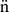
ve Bayesian classifier implemented in RDPClassifier ver. 2.5 [23], Barcoder and ConstrainedNJ implemented in SAP ver. 1.0.6 [29], and the 1-NN, 5-NN, 97%-NN, 99%-NN, NNCauto, and QCauto methods implemented in Claident ver. 0.1.2012.06.16 were measured and compared by no-leave-one-out cross-validation (no-LOOCV) and leave-one-out cross-validation (LOOCV). These methods represented composition-based, similarity-based, and hybrid approaches. The selection of the methods was based on assignment speed, machine-learning speed, and the limitation of computing resources (e.g., amount of memory). Programs based on a tree-based approach were not applicable to the benchmark due to their prerequisites. NCBI BLAST ver. 2.2.26 [24] and NCBI BLAST+ 2.2.26+ [34] were used in SAP and Claident, respectively.

The taxonomic assignment of the above-mentioned queries was conducted using the local reference sequence databases of each organismal group. In the benchmark based on no-LOOCV, all query sequences were retained in the corresponding local database. Therefore, no-LOOCV simulates the situation in which the query sequence is known and has been deposited on reference databases. In contrast, each query sequence was removed from the corresponding reference database in the LOOCV: this setting simulates the situation in which the query is unknown sequence. The minimum global alignment similarity of the best BLAST hit was set to 0.5 in the analyses with Barcoder and ConstrainedNJ because no similar sequences were retrieved when the default value was used. For the 97%-NN and 99%-NN methods, the maximum number of nearest-neighbor sequences was set to be 100. For all the other parameters, the default settings of each program were used. Both the full-length and the 200-bp-long mini-barcode query settings were used in this benchmark. Because the reference sequence databases of animal *COX1*, bacterial/archaeal 16S, and fungal ITS were too large to run LOOCV for RDPClassifier, the LOOCV was not used in the program for the barcode loci.

#### Summarizing the Benchmark

To summarize the benchmark results, the number of correctly identified taxonomic levels was used as an index representing the degree of correctness of taxonomic assignment. This correctness index has the maximum value 6 when the taxonomic information at all the phylum/division, class, order, family, genus, and species levels is correctly assigned, whereas the index has the minimum value 0 when taxonomic information at all the six taxonomic levels is erroneously assigned to a query or a query remained unidentified even at the phylum/division level. However, the correctness index alone does not fully depict the success/failure of taxonomic identification because a low correctness score provides no information on whether a query is assigned to an incorrect taxon (i.e., misidentified) or it is unidentified due to the lack of similar DNA sequences in a reference database.

Therefore, we also measured the degree of misidentification caused by each method. The number of incorrectly identified taxonomic levels was used for this purpose. This incorrectness index has the maximum value 6 when the taxonomic assignment of all the six taxonomic levels is incorrect. Meanwhile, the index has the minimum value 0 when the taxonomic assignment does not return incorrect results at any taxonomic level; note that this included the situation in which a query is unidentified even at the phylum/division level. The frequency of queries with their respective correctness or incorrectness values (scores) was determined for each method for each of the animal *COX1*, bacterial/archaeal 16S, fungal ITS, plant *matK*, plant *rbcL*, and plant *trnH*-*psbA* regions.

Several previous studies used accuracy, sensitivity (a.k.a. recall rate), and/or specificity for comparing the performance of taxonomic assignment among different methods [21], [38]. However, these indices need to be calculated at each taxonomic level. On the other hand, by using correctness and incorrectness indices, we can evaluate to what degree the queries are successfully identified, misidentified, or unidentified, taking identification results at all the examined taxonomic levels into account. Detailed benchmark results at each taxonomic level are provided as supplementary ones. In the supplementary analysis, the frequencies of correctly identified queries, incorrectly identified queries, and unidentified queries were determined at each taxonomic level for each barcode locus. Unidentified queries were further classified into two categories: queries unidentified at the focal taxonomic level and incorrectly identified at higher taxonomic levels, and queries unidentified at the focal level but not incorrectly identified at higher levels.

## Results

### Characteristics of Constructed Reference Databases and Query Sequences

From the NCBI nt sequence database, local reference sequence databases were constructed with 608,412 animal *COX1*, 338,405 bacterial/archaeal 16S, 147,695 fungal ITS, 43,555 plant *matK*, 53,573 plant *rbcL*, and 11,714 plant *trnH*-*psbA* sequences. The numbers of query sequences (genera) were 3,714 for animal *COX1*, 1,642 for bacterial/archaeal 16S, 1,073 for fungal ITS, and 3,012 for plant *matK*, 3,754 for plant *rbcL*, and 1,262 for plant *trnH*-*psbA*. All the local reference sequence data sets and the query sequences are available as Datasets S1, S2, S3, S4, S5, S6, S7, S8, S9, S10, S11, S12, S13, S14, S15, S16, S17, S18.

### No-Leave-One-Out Cross-Validation

**Figure 3:**
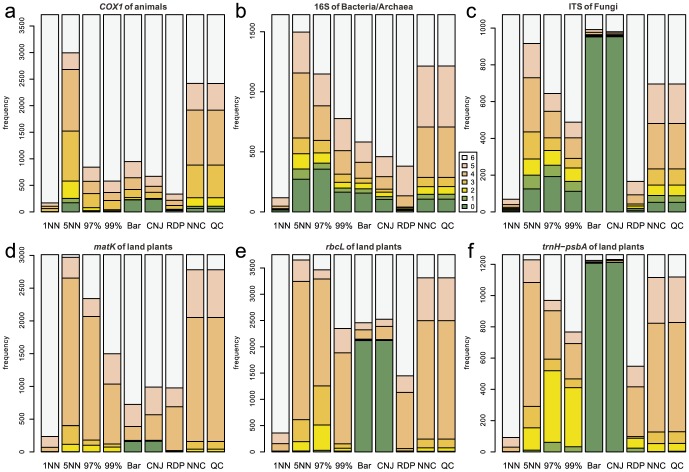
Frequencies of correctness scores in the no-LOOCV of full-length query sets. The number of correctly identified taxonomic levels is used as an index representing the degree of correctness of taxonomic assignment. This correctness index has the maximum value 6 when the taxonomic information at all the phylum/division, class, order, family, genus, and species levels is correctly assigned. On the other hand, the index has the minimum value 0 when taxonomic information at all the six taxonomic levels is erroneously assigned to a query or a query remains unidentified even at the phylum/division level. 1NN, 5NN, 97%, 99%, Bar, CNJ, RDP, NNC, and QC means 1-NN, 5-NN, 97%-NN, 99%-NN, Barcoder, ConstrainedNJ, RDPClassifier, NNCauto, and QCauto methods, respectively. (a) Animal *COX1*. (b) Bacterial/Archaeal 16S. (c) Fungal ITS. (d) Plant *matK*. (e) Plant *rbcL*. (f) Plant *trnH*-*psbA*.

**Figure 4:**
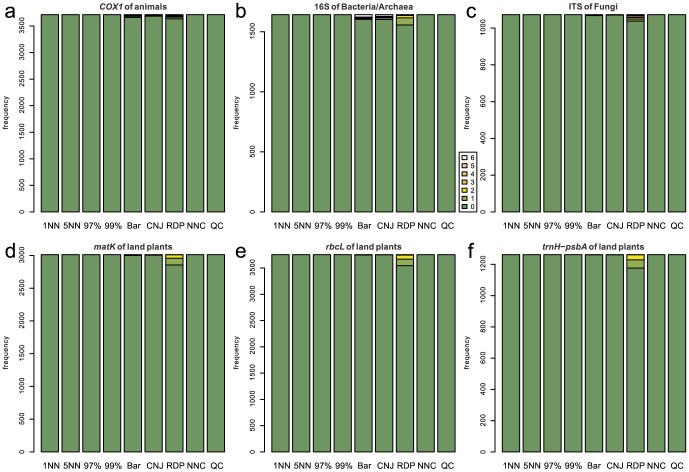
Frequencies of incorrectness scores in the no-LOOCV of full-length query sets. The number of incorrectly identified taxonomic levels is used as an index representing the degree of incorrectness of taxonomic assignment. This incorrectness index has the maximum value 6 when the taxonomic assignment of all the six taxonomic levels is incorrect. On the other hand, the index has the minimum value 0 when the taxonomic assignment does not return incorrect results at any taxonomic level: note that this includes the situation in which a query is unidentified even at the phylum/division level. 1NN, 5NN, 97%, 99%, Bar, CNJ, RDP, NNC, and QC represent the 1-NN, 5-NN, 97%-NN, 99%-NN, Barcoder, ConstrainedNJ, RDPClassifier, NNCauto, and QCauto methods, respectively. (a) Animal *COX1*. (b) Bacterial/Archaeal 16S. (c) Fungal ITS. (d) Plant *matK*. (e) Plant *rbcL*. (f) Plant *trnH*-*psbA*.

As expected from the definition of no-LOOCV, high (5–6) correctness scores were observed most frequently for the 1-NN method at all the examined barcode loci in this type of cross-validation (Fig. 3). In the taxonomic assignment using fungal ITS, plant *rbcL*, and plant *trnH*-*psbA*, Barcoder and ConstrainedNJ displayed a high proportion of very low (0–2) correctness scores (Fig. 3c, e, and f). No clear difference was observed regarding the incorrectness scores among the nine methods for all the examined barcode loci (Fig. 4). However, RDPClassifier, Barcoder, and ConstrainedNJ occasionally returned incorrect taxonomic information.

### Leave-One-Out Cross-Validation

**Figure 5:**
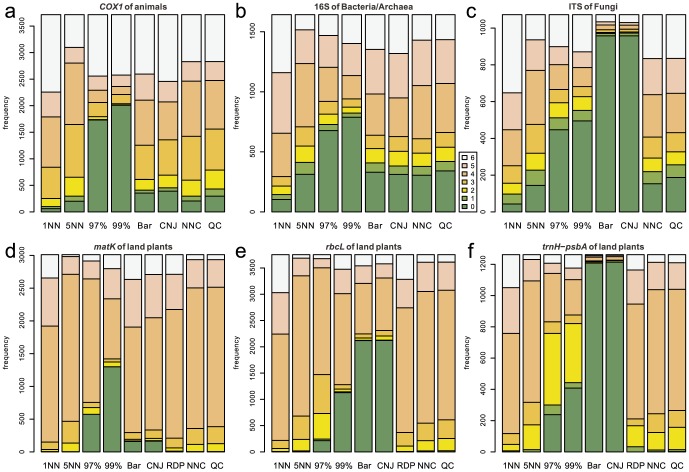
Frequencies of correctness scores in the LOOCV of full-length query sets. 1NN, 5NN, 97%, 99%, Bar, CNJ, RDP, NNC, and QC represent the 1-NN, 5-NN, 97%-NN, 99%-NN, Barcoder, ConstrainedNJ, RDPClassifier, NNCauto, and QCauto methods, respectively. (a) Animal *COX1*. (b) Bacterial/Archaeal 16S. (c) Fungal ITS. (d) Plant *matK*. (e) Plant *rbcL*. (f) Plant *trnH*-*psbA*. See the caption of Fig. 3 for the explanation of the correctness index.

**Figure 6:**
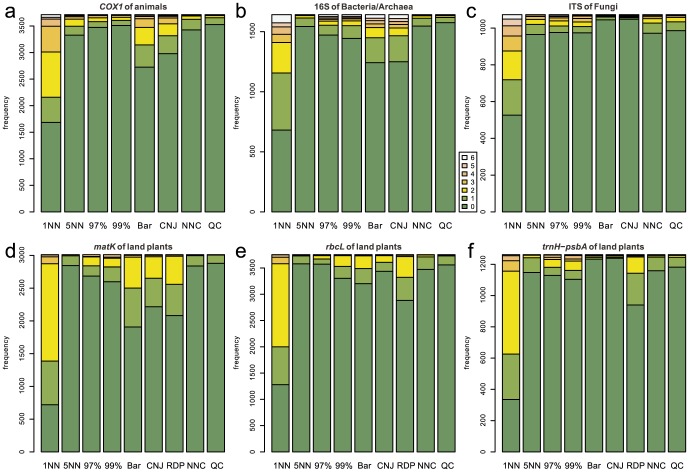
Frequencies of incorrectness scores in the LOOCV of full-length query sets. 1NN, 5NN, 97%, 99%, Bar, CNJ, RDP, NNC, and QC represent the 1-NN, 5-NN, 97%-NN, 99%-NN, Barcoder, ConstrainedNJ, RDPClassifier, NNCauto, and QCauto methods, respectively. (a) Animal *COX1*. (b) Bacterial/Archaeal 16S. (c) Fungal ITS. (d) Plant *matK*. (e) Plant *rbcL*. (f) Plant *trnH*-*psbA*. See the caption of Fig. 4 for the explanation of the incorrectness index.

In the LOOCV, the 1-NN method displayed high (5–6) correctness scores as shown in the no-LOOCV (Fig. 5), but the taxonomic assignment by this method resulted in a remarkably high proportion of misidentification (Fig. 6). The 97%-NN and 99%-NN methods displayed low (0–2) correctness scores most frequently for animal *COX1*, bacterial/archaeal 16S, and plant *matK* (Fig. 5a, b, and d). Likewise, Barcoder and ConstrainedNJ displayed low correctness scores for fungal ITS, plant *rbcL*, and plant *trnH*-*psbA* (Fig. 5c, e, and f). The results of the 5-NN, NNCauto, and QCauto methods were similar to each other, but the NNCauto and QCauto methods were more conservative than 5-NN when they were evaluated by the incorrectness index (Figs. 5 and 6). Between the two new methods, the QCauto method returned more conservative results than the NNCauto method (Figs. 5 and 6).

### Detailed Benchmark Results

Overall, qualitatively and quantitatively similar results were obtained under the full-length (Figs. 3, 4, 5, 6) and mini-barcode (Figs. S1, S2, S3, S4) settings based on both the no-LOOCV and LOOCV. Detailed results of full-length query benchmarks for each barcode locus at each taxonomic level are shown in Figs. S5 and S6.

## Discussion

In the benchmark based on no-LOOCV, the 1-NN method most frequently returned perfect identification results (i.e., correctness = 6) of all the methods tested for all the barcode loci (Fig. 3). Given that a query sequence was not removed from a reference sequence database in a no-LOOCV, this result suggests that the 1-NN method is the best DNA barcoding method, if the barcode sequences of all potentially observable species are registered to a reference database. However, in the LOOCV, the 1-NN method returned erroneous identification results (i.e., high incorrectness scores) most frequently among the examined methods (Fig. 6). As the LOOCV simulates the situation in which DNA samples contain undescribed or poorly investigated species/taxa, the observed frequency of unsuccessful taxonomic identifications by the 1-NN method is a serious concern. Taking into account that the sequence databases of most barcode loci do not contain all species in their target taxonomic groups (e.g., [11]), the 1-NN method could lead to severe misidentification in DNA barcoding.

In the LOOCV, the 97%-NN and 99%-NN methods produced low correctness scores compared to the other methods, especially for the taxonomic identification of animal *COX1*, bacterial/archaeal 16S, and plant *matK* loci (Fig. 5). This type of method using identity-cutoff values often fails to find sequences similar to a query, thereby leaving a high proportion of queries “unidentified” (Fig. S6). Furthermore, these *n*%-NN methods should be used with caution because they can result in high rates of misidentification (incorrectness >3) for such barcode loci as bacterial/archaeal 16S and plant *trnH*-*psbA* loci compared to our new methods (Fig. 6).

RDPClassifier, a composition-based approach, displayed high correctness scores in the no-LOOCV (Fig. 3). Thus, as with the 1-NN methods, a composition-based approach potentially enables efficient taxonomic identification if a reference sequence database includes sequences of all the potentially observable species. However, RDPClassifier returned erroneous identification results most frequently of all the methods in the no-LOOCV for all the examined barcode loci (Fig. 4). Moreover, in the LOOCV, this composition-based approach returned nonzero incorrectness scores frequently for all of the plant barcode loci examined (Fig. 6d–f).

The hybrid of similarity-based and tree-based approaches, which was implemented as Barcoder and ConstrainedNJ methods in SAP, produced very low rates of successful identification for fungal ITS and plant *trnH*-*psbA* loci in both the no-LOOCV and LOOCV (Figs. 3c, 3f, 5c, and 5f). Given that these loci display considerably high variation in their sequence lengths, the difficulty in achieving multiple sequence alignments may be responsible for the high proportion of incorrect taxonomic assignment by Barcoder and ConstrainedNJ. These two methods also often failed to achieve taxonomic identification in the DNA barcoding based on plant *rbcL* (Figs. 3e and 5e). As the tree-based part of the Barcoder and ConstrainedNJ methods required at least 95% node support by default, the low interspecific sequence variation in the plant *rbcL* gene presumably hampered taxonomic identification by the locus.

Among the methods based on a similarity-based approach, the 5-NN, NNCauto, and QCauto methods were characterized by a low frequency of misidentification in the LOOCV (Fig. 6) and intermediate degrees of correct identification in both the no-LOOCV and LOOCV (Figs. 3 and 4). Of the similarity-based methods, the QCauto method had identification success rates comparable to the 5-NN and NNCauto methods (Fig. 5) and displayed a lower proportion of misidentification compared to the remaining two methods (Fig. 6). The QCauto method was therefore the most conservative choice among the examined methods. Meanwhile, the QCauto method requires a longer computational time than the 5-NN and NNCauto methods. Given that the 5-NN method enables fast execution because of the simplicity of its algorithm, it may be suitable for the rapid processing of a large number of query sequences. Otherwise, the QCauto method is ideal because it enables more accurate taxonomic identification. However, great care should be taken in applying the 5-NN method to empirical work because no firm theoretical background exists for retrieving a fixed number of similar sequences in the course of DNA barcoding. Therefore, the NNCauto method could be a good alternative to the QCauto method because its similar sequence search process has a strictly defined theoretical background like the QCauto method (Fig. 2), but it is less computationally intensive than the QCauto method.

Overall, the proportion of queries that were successfully identified to genus or species level (i.e., correctness score = 5 or 6) was less than 50% for most combinations of the DNA barcoding methods and genetic loci in the LOOCV (Fig. 5). For all the methods examined in this study, the failure of taxonomic identification resulted mainly from the absence of reference sequences similar to the queries (“unidentified”) rather than misidentification (“incorrectly identified”; Fig. S6). Thus, increasing the number of reference sequences as well as enhancing the taxon coverage of the reference databases is of particular importance to increase the efficiency of DNA barcoding. Moreover, in a similarity-based approach, relaxing the settings of the LCA-algorithm-based taxon assignment could reduce the proportion of “unidentified” queries. Basically, the LCA algorithm is very stringent in that it allows identification at a taxonomic level only when the taxonomic information of all similar sequences are consistent with each other [25]. Because reference sequence databases contain many misidentified sequences, the stringency of the LCA algorithm may produce “unidentified” results. Therefore, by tolerating a small proportion of similar sequences whose taxonomic information is inconsistent with that of the remaining similar sequences, the proportion of unidentified queries may be reduced to some extent. Although the newly developped progam Claident implements this “relaxed-LCA” algorithm, the degree of such relaxation should be optimized by users by performing independent runs with different relaxation parameter values.

The causes of misidentification in the benchmark analysis can be classified into five main categories: 1) the error of the taxonomic information in used query sequences, 2) the error of the taxonomic information in used reference sequences, 3) the use of inappropriate sequence similarity indices, 4) the application of inappropriate criteria in retrieving similar sequences, and 5) a discrepancy between the taxonomic system and phylogenetic information. Categories 1 and 2 result in apparent misidentification, wherein the proportion of successful taxonomic assignments by each method is underestimated. In contrast, categories 3 and 4 result in actual misidentification, wherein the low proportion of successful identification in a benchmark illuminates the need to improve reference sequence databases and/or DNA barcoding methodologies. For category 5, any discrepancy between a taxonomic system and the phylogeny of a used barcoding locus can result in misidentification in a benchmark. For example, ancestral polymorphisms in a barcode locus and subsequent incomplete lineage sorting can cause the sharing of multiple identical alleles among sister species, thereby hampering taxonomic identification with the barcode locus at species level [33], [39], [40].

To reduce misidentification in the categories 1 and 2, erroneous taxonomic information in public nucleotide databases should be corrected. Alternatively, the proportion of misidentification may be reduced by using qualified databases that include the sequences of the specimens identified by experienced experts. Databases such as BOLD [41], SILVA [42], and UNITE [43] potentially provide a basis for reliable taxonomic identification by means of DNA barcoding, but the number of sequences registered to these databases remains quite small. With regard to sequence identity indices (category 3), we herein used BLAST raw scores based on the local alignment similarity provided by BLAST+ for the taxonomic assignment of the *k*-NN, *n*%-NN, NNCauto, and QCauto methods. The application of global-alignment similarity instead of local-alignment similarity may improve identification performance as previously reported for the 1-NN method [44], although calculating global-alignment similarity is computationally much more intensive. To reduce the category 4 misidentification, the application of the QCauto (or NNCauto) method is recommended as detailed above. Finally, to reduce misidentification under category 5 conditions, we need to adopt multiple strategies. For example, the hierarchical structure of taxonomy should be reexamined, especially for clades that have recently undergone adaptive radiation. In addition, for the species described by polyphyletic or paraphyletic lineages, haplotypes of respective monophyletic lineages need to be registered to reference sequence databases.

Intriguingly, misidentification was more frequent at genus level than at all the other levels for all barcode loci in the LOOCV (Fig. S6). While genus name is essential for the description of a novel species, higher-level taxonomy can be left unresolved. Therefore, a discrepancy between a taxonomic system and phylogenetic information can be most frequently caused at genus level, and the discrepancy may induce erroneous taxonomic information of reference sequences, resulting in relatively high misidentification rates at genus level in the LOOCV. Thus, a detailed investigation of our benchmark results will help to recognize the characteristics of current taxonomic systems.

Several existing benchmark studies of DNA barcoding are based on simulations with artificially generated data sets [45], [46] or with real database sequences of a single organismal taxon [45], [47]. We reported herein the benchmark results of a wide variety of existing methods and novel DNA barcoding methods by using the existing sequences of bacteria, archaea, animals, fungi, and land plants. As detailed above, further improvements in identification algorithms as well as the quantitative/qualitative enhancements of reference sequence databases are required to promote taxonomic, evolutionary, and ecological studies of diverse organisms by means of high-throughput DNA barcoding.

## Supporting Information

Dataset S1 **Reference sequence sets of animal** ***COX1*** **.** Nucleotide sequences used as reference sets of animal *COX1*.
(ZIP)

Dataset S2 **Reference sequence sets of bacterial/archaeal 16S.** Nucleotide sequences used as reference sets of bacterial/archaeal 16S.
(ZIP)

Dataset S3 **Reference sequence sets of fungal ITS.** Nucleotide sequences used as reference sets of fungal ITS.
(ZIP)

Dataset S4 **Reference sequence sets of** ***matK*** **of land plants.** Nucleotide sequences used as reference sets of *matK* of land plants.
(ZIP)

Dataset S5 **Reference sequence sets of** ***rbcL*** **of land plants.** Nucleotide sequences used as reference sets of *rbcL* of land plants.
(ZIP)

Dataset S6 **Reference sequence sets of** ***trnH*** **-** ***psbA*** **of land plants.** Nucleotide sequences used as reference sets of *trnH*-*psbA* of land plants.
(ZIP)

Dataset S7 **Full-length query sequence sets of animal** ***COX1*** **.** Nucleotide sequences used as full-length query sets of animal *COX1*.
(ZIP)

Dataset S8 **Full-length query sequence sets of bacterial/archaeal 16S.** Nucleotide sequences used as full-length query sets of bacterial/archaeal 16S.
(ZIP)

Dataset S9 **Full-length query sequence sets of fungal ITS.** Nucleotide sequences used as full-length query sets of fungal ITS.
(ZIP)

Dataset S10 **Full-length query sequence sets of** ***matK*** **of land plants.** Nucleotide sequences used as full-length query sets of *matK* of land plants.
(ZIP)

Dataset S11 **Full-length query sequence sets of** ***rbcL*** **of land plants.** Nucleotide sequences used as full-length query sets of *rbcL* of land plants.
(ZIP)

Dataset S12 **Full-length query sequence sets of** ***trnH*** **-** ***psbA*** **of land plants.** Nucleotide sequences used as full-length query sets of *trnH*-*psbA* of land plants.
(ZIP)

Dataset S13 **Mini-barcode query sequence sets of animal** ***COX1*** **.** Nucleotide sequences used as mini-barcode query sets of animal *COX1*.
(ZIP)

Dataset S14 **Mini-barcode query sequence sets of bacterial/archaeal 16S.** Nucleotide sequences used as mini-barcode query sets of bacterial/archaeal 16S.
(ZIP)

Dataset S15 **Mini-barcode query sequence sets of fungal ITS.** Nucleotide sequences used as mini-barcode query sets of fungal ITS.
(ZIP)

Dataset S16 **Mini-barcode query sequence sets of** ***matK*** **of land plants.** Nucleotide sequences used as mini-barcode query sets of *matK* of land plants.
(ZIP)

Dataset S17 **Mini-barcode query sequence sets of** ***rbcL*** **of land plants.** Nucleotide sequences used as mini-barcode query sets of *rbcL* of land plants.
(ZIP)

Dataset S18 **Mini-barcode query sequence sets of** ***trnH*** **-** ***psbA*** **of land plants.** Nucleotide sequences used as mini-barcode query sets of *trnH*-*psbA* of land plants.
(ZIP)

Figure S1 **Frequencies of correctness scores in the no-LOOCV of mini-barcode query sets.** The number of correctly identified taxonomic levels is used as an index representing the degree of correctness of taxonomic assignment. This correctness index has the maximum value 6 when the taxonomic information at all the phylum/division, class, order, family, genus, and species levels is correctly assigned. On the other hand, the index has the minimum value 0 when taxonomic information at all the six taxonomic levels is erroneously assigned to a query or a query remains unidentified even at the phylum/division level. 1NN, 5NN, 97%, 99%, Bar, CNJ, RDP, NNC, and QC represent the 1-NN, 5-NN, 97%-NN, 99%-NN, Barcoder, ConstrainedNJ, RDPClassifier, NNCauto, and QCauto methods, respectively. (a) Animal *COX1*. (b) Bacterial/Archaeal 16S. (c) Fungal ITS. (d) Plant *matK*. (e) Plant *rbcL*. (f) Plant *trnH*-*psbA*.
(EPS)

Figure S2 **Frequencies of incorrectness scores in the no-LOOCV of mini-barcode query sets.** The number of incorrectly identified taxonomic levels is used as an index representing the degree of incorrectness of taxonomic assignment. This incorrectness index has the maximum value 6 when the taxonomic assignment of all the six taxonomic levels is incorrect. On the other hand, the index has the minimum value 0 when the taxonomic assignment does not return incorrect results at any taxonomic level: note that this includes the situation in which a query is unidentified even at the phylum/division level. 1NN, 5NN, 97%, 99%, Bar, CNJ, RDP, NNC, and QC represent the 1-NN, 5-NN, 97%-NN, 99%-NN, Barcoder, ConstrainedNJ, RDPClassifier, NNCauto, and QCauto methods, respectively. (a) Animal *COX1*. (b) Bacterial/Archaeal 16S. (c) Fungal ITS. (d) Plant *matK*. (e) Plant *rbcL*. (f) Plant *trnH*-*psbA*.
(EPS)

Figure S3 **Frequencies of correctness scores in the LOOCV of mini-barcode query sets.** 1NN, 5NN, 97%, 99%, Bar, CNJ, RDP, NNC, and QC represent the 1-NN, 5-NN, 97%-NN, 99%-NN, Barcoder, ConstrainedNJ, RDPClassifier, NNCauto, and QCauto methods, respectively. (a) Animal *COX1*. (b) Bacterial/Archaeal 16S. (c) Fungal ITS. (d) Plant *matK*. (e) Plant *rbcL*. (f) Plant *trnH*-*psbA*. See the caption of Fig. S1 for the explanation of the correctness index.
(EPS)

Figure S4 **Frequencies of incorrectness scores in the LOOCV of mini-barcode query sets.** 1NN, 5NN, 97%, 99%, Bar, CNJ, RDP, NNC, and QC represent the 1-NN, 5-NN, 97%-NN, 99%-NN, Barcoder, ConstrainedNJ, RDPClassifier, NNCauto, and QCauto methods, respectively. (a) Animal *COX1*. (b) Bacterial/Archaeal 16S. (c) Fungal ITS. (d) Plant *matK*. (e) Plant *rbcL*. (f) Plant *trnH*-*psbA*. See the caption of Figure S2 for the explanation of the incorrectness index.
(EPS)

Figure S5 **Frequencies of “correctly identified at the focal level”, “incorrectly identified at the focal level”, “unidentified at the focal level but incorrectly identified at higher level”, and “unidentified at the focal level and correctly identified at higher level” in the no-LOOCV of full-length query sets.** 1NN, 5NN, 97%, 99%, Bar, CNJ, RDP, NNC, and QC represent the 1-NN, 5-NN, 97%-NN, 99%-NN, Barcoder, ConstrainedNJ, RDPClassifier, NNCauto, and QCauto methods, respectively.
(EPS)

Figure S6 **Frequencies of “correctly identified at the focal level”, “incorrectly identified at the focal level”, “unidentified at the focal level but incorrectly identified at higher level”, and “unidentified at the focal level and correctly identified at higher level” in the LOOCV of full-length query sets.** 1NN, 5NN, 97%, 99%, Bar, CNJ, RDP, NNC, and QC represent the 1-NN, 5-NN, 97%-NN, 99%-NN, Barcoder, ConstrainedNJ, RDPClassifier, NNCauto, and QCauto methods, respectively.
(EPS)

Table S1 **Used search keywords for retrieving the GenBank IDs of the sequences of respective barcode loci.**
(CSV)
