# Correction: Two New Computational Methods for Universal DNA Barcoding: A Benchmark Using Barcode Sequences of Bacteria, Archaea, Animals, Fungi, and Land Plants

**DOI:** 10.1371/journal.pone.0152242

**Published:** 2016-03-24

**Authors:** Akifumi S. Tanabe, Hirokazu Toju

In the third sentence of the first paragraph of sub-subsection “Query-Centric Auto-*k*-Nearest-Neighbor (QCauto) Method”, within the Materials and Methods section, the variable of expression is incorrect. The correct sentence should read: A BLAST-search of reference sequences similar to the query was then performed, and subsequently, all the neighborhood sequences (Ns) whose distance to the query sequences was equal to or smaller than *D*_QB_ were retrieved (i.e., *D*_QB_ ≥ *D*_QN_; Fig. 2d).
